# Supplementary material for: Learning how network structure shapes decision-making for bio-inspired computing
Source: Nat Commun. 2023 May 23;14:2963. doi: 10.1038/s41467-023-38626-y (PMC10206104; doi:10.1038/s41467-023-38626-y)
Supplement: Supplementary file 1 — Supplementary Information [file 41467_2023_38626_MOESM1_ESM.pdf]

Learning how network structure shapes decision-making for bio-inspired computing

Michael Schirner[1,2,3,4,5,*], Gustavo Deco[6,7,8,9] & Petra Ritter[1,2,3,4,5,*]

[1]Berlin Institute of Health at Charité, Universitätsmedizin Berlin, Charitéplatz 1, Berlin 10117, Germany
[2]Department of Neurology with Experimental Neurology, Charité, Universitätsmedizin Berlin, Corporate member of Freie Universität Berlin and Humboldt Universität zu Berlin, Charitéplatz 1, Berlin 10117, Germany
[3]Bernstein Focus State Dependencies of Learning and Bernstein Center for Computational Neuroscience, Berlin, Germany
[4]Einstein Center for Neuroscience Berlin, Charitéplatz 1, Berlin 10117, Germany
[5]Einstein Center Digital Future, Wilhelmstraße 67, Berlin 10117, Germany
[6]Department of Information and Communication Technologies, Center for Brain and Cognition, Computational Neuroscience Group, Universitat Pompeu Fabra, Barcelona, Spain
[7]Institució Catalana de la Recerca i Estudis Avançats, Barcelona, Spain
[8]Department of Neuropsychology, Max Planck Institute for Human Cognitive and Brain Sciences, Leipzig, Germany
[9]School of Psychological Sciences, Turner Institute for Brain and Mental Health, Monash University, Melbourne, Clayton, Australia
*Corresponding authors (michael.schirner@bih-charite.de, petra.ritter@bih-charite.de)

SUPPLEMENTARY INFORMATION

Supplementary Tables

Quantity	Value	Description
State variables		
$r_i^{(E,I)}$		Population firing rate of the excitatory (E) or inhibitory (I) population in brain area *i*
$S_i^{(E,I)}$		NMDA (E) and GABA (I) synaptic gating
$I_i^{(E,I)}$		Sum of input currents
v_i		Noise sampled from the standard normal distribution
Parameters		
I_0	0.382 nA	Overall effective external input
$W_{(E,I)}$	1 (E); 0.7 (I)	Modulation of I_0 for excitatory, respectively, inhibitory population
w_+	1.4	Local excitatory recurrence
J_{NMDA}	0.15 nA	Excitatory synaptic coupling
J_i	Obtained by FIC	Feedback inhibitory synaptic coupling
C_{ij}	Obtained by dwMRI tractography	Structural connectivity matrix
w_{ij}^{LRE}	Obtained by FC fitting	Long-range excitation
w_{ij}^{FFI}	Obtained by FC fitting	Feedforward inhibition
a_E, b_E, d_E	310 (1/nC), 125 (Hz), 0.16 (s)	Parameters of excitatory population's frequency-current (f-I) function
a_I, b_I, d_I	615 (1/nC), 177 (Hz), 0.087 (s)	Parameters of inhibitory population's f-I function
γ_E, γ_I	6.41×10^{-4}, 1.0×10^{-3}	Rate of saturation
τ_E, τ_I	100 ms, 10 ms	Time scales of synaptic activity
σ	0.01	Noise scaling

Supplementary Table 1. State variables and parameters of the large-scale BNM.

**Quantity**	**Value**	**Description**
State variables		
r		Population firing rates
S_i^n		NMDA synaptic gating
I_i^n		Sum of input currents
$I_{noise,i}$		Noise current
Parameters		
I_0	0.334 nA	Overall effective external input
$J_{i,j}^{m\to n}$	$J_{\{AA,BB\}}^{PPC\to PPC} = 0.3169$<br>$J_{\{AB,BA\}}^{PPC\to PPC} = -0.0331$<br>$J_{\{AA,BB\}}^{PFC\to PFC} = 0.351$<br>$J_{\{AB,BA\}}^{PFC\to PFC} = -0.0671$<br>$J_{\{AA,AB\}}^{PPC\to PFC} = 0.02$<br>$J_{\{AA,AB\}}^{PFC\to PPC} = 0.075$	Connection weight from population j in module m to population i in module n
a, b, c	270 (1/nC), 108 (Hz), 0.154 (s)	Parameters of frequency-current (f-I) function
γ	6.41×10^{-4}	Kinetic parameters
τ	60 ms	Time scales of synaptic activity

Supplementary Table 2. State variables and parameters of the DM circuit.

Supplementary Notes

How E/I-ratios control synchrony and amplitude of synaptic currents

Why did E/I-ratios modulate population synchronization? The E/I-ratio of each long-range connection was set by the two parameters w^{LRE} and w^{FFI} (Equations 1, 2). To increase the E/I-ratio w^{LRE} has been increased and w^{FFI} has been decreased (Fig. 4c). As w^{LRE} was increased the excitatory population activity depended more on long-range inputs and less on local inputs and noise: long-range inputs increasingly dominated the activity of excitatory populations, which entrained the nodes towards higher synchrony. Vice versa, when w^{FFI} was increased inhibitory population activity became increasingly synchronized with the global inputs and, as it is inhibitory activity, it drove its excitatory partner towards the opposite dynamics, which lead to anti-synchronization of the excitatory population with the global input. Interestingly, at a balanced E/I-ratio these tendencies towards synchronization versus antisynchronization were also balanced--they canceled each other--and hence absolute correlations were at their minima (close to zero) for balanced E/I-ratios (Figs. 4d, e).

To better understand the correlation of simulated synaptic currents with the participants' empirical PMAT24_A_RTCR (Fig. 4a, b; Supplementary Fig. 2) we studied synaptic currents in dependence of the E/I-ratio in the two-node model (Fig. 4f, g). One important observation is that FIC had reversed the relationship between E/I-ratios and input amplitudes: without FIC the input amplitude had monotonically increased for increasing E/I-ratios (Fig. 4f, blue curve); in contrast, when FIC was active the input amplitude peaked at a relatively low E/I-ratio and from there on only decreased for increasing E/I-ratios (Fig. 4f, black curve). This observation is important, because it shows that FIC was required to simultaneously obtain a direct relationship between E/I-ratios and FC and an indirect relationship between input amplitude and RTs. In contrast, without FIC, the models with a higher FC would have had increased input amplitudes. As we show here, this no-FIC scenario is implausible, because increased amplitudes would have increased the speed of decision-making, and not slowed it down.

The observation that increased E/I-ratios had led to decreased current amplitudes is non-trivial: if excitatory coupling was increased and inhibitory coupling decreased, why did the input amplitude decrease when FIC was active, instead of increase, as it did without FIC (black versus blue curve in Fig. 4f)? Our explanation is based on the observation that the variance and the mean of synaptic inputs appear to be coupled through FIC as indicated by comparing Fig. 4f and Fig. 4g: the black curves show inverted shapes while the blue curves appear unrelated. To test this hypothesis more directly, we injected one pair of coupled excitatory and inhibitory population models with a pure 10 Hz sine wave with increasing variances and compared outcomes with FIC versus without FIC (Fig. 4h-j). Plotting variance against mean (Fig. 4h) shows that FIC compensates for higher variances by decreasing the mean of inputs in order to stabilize firing: as the variance of inputs had been increased by increasing E/I-ratios (using the multiplicative parameters w^{LRE} and w^{FFI} in Equations 1, 2), it was necessary for FIC to decrease its mean amplitude in order to obtain an average population firing rate at the set target value. This was necessary, because the peaks of the firing activity continued to grow for increasing input variance, while the troughs were bound from below due to physiological constraints: firing could not get below 0 Hz and it was therefore necessary for FIC to decrease the mean input, which prolonged the inhibition time window to arrive at the desired firing rate (compare traces with FIC in Fig. 4i versus traces without FIC in Fig. 4j). Such a rectification of neuronal oscillations due to shunting inhibitory feedback was also identified as the underlying mechanism for the pulsatile inhibitory action of alpha rhythms[79].

Studying DM and WM with a frontoparietal circuit model

To more directly study the modulatory effects of input amplitude and correlation on cognitive performance we adopted a frontoparietal circuit model for WM and DM[37], which we call DM circuit here. The DM circuit attractor network uses recurrent excitation to enable stimulus-selective persistent activity for WM[84] and winner-take-all competition for DM[77]. The DM circuit consists of four mutually and recurrently coupled neural mass models: two located in prefrontal cortex (PFC) and two in posterior parietal cortex (PPC). The two population pairs encode the outcomes of a binary decision, respectively, two working memory states. For WM, recurrent excitation creates a bistability between

low- and high-activity attractors, enabling to encode memory states. Cross-inhibition between populations that encode opposing outcomes leads to winner-take-all competition; recurrent excitation generates ramping activity through slow reverberation, which allows integration of evidence until one of the two attractors is reached[37]. Evidence for either of the two options is simulated by constant currents to the two PPC populations with one population receiving a slightly higher current. A decision is made, respectively, a WM state is assumed, when one of the two populations in PFC reaches the high-activity attractor, following sufficient integration of inputs from PPC. The robustness of a WM state is estimated by applying a current to the other population pair as a distractor. The ongoing WM state is robust, if the encoding population pair continues its persistent high-firing activity after the distractor current is removed again. For DM, a correct decision is formed when the PPC and PFC population pair that corresponds to the preferred option reaches the high-activity attractor state, while the other population pair is inhibited and in a low-firing state (see Supplementary Fig. 4a for exemplary time series of correct versus incorrect decisions). If the difference in evidence between the two options gets smaller, that is, if the associated evidence currents differ only slightly in amplitude, it gets increasingly likely that brain network background activity (in DM circuit modelled by noise) triggers incorrect decisions, because the wrong population is accidentally brought over the separatrix that separates the two basins of attraction of the respective attractors.

How input amplitude modulates DM performance

To study the mechanism how input amplitude switches between fast-but-faulty and precise-but-slow modes of decision-making (Fig. 5a, b) we analyzed the phase space of the DM circuit for different input amplitudes (Supplementary Fig. 4). Specifically, we compared phase portraits for five different PPC input amplitude offsets (ranging from -0.008 *nA* to 0.004 *nA*) and three time points of interest during which feedback from PFC increased until decisions were made. To simulate difficult decisions the PPC populations were stimulated with almost identical evidence currents, that is, the supplied evidence had a low contrast. Supplementary Fig. 4a shows exemplary firing rate time series for all four DM populations that only differed with respect to the five tested input amplitudes. In this example, the three trials with low input amplitude resulted in correct decisions, while the two trials with high input amplitude resulted in incorrect decisions. The accompanying phase portraits (Supplementary Fig. 4b) show how phase flow moves the state from the origin into the direction of the attractors. The two stable attractors correspond to the two possible outcomes of the decision-making with one of the population pairs assuming a persistent high-firing state and the other one assuming a low-activity state due to cross-inhibition. In these experiments the PPC population A received the higher current, therefore, the attractor at the bottom right represents a correct decision and the attractor at the top left represents an incorrect decision.

Analyzing the phase portraits (Supplementary Fig. 4b) shows that input amplitude had a considerable impact on the system's flow. For the DM circuit default configuration with an input offset of 0 *nA* (middle column) a separatrix divided the phase space diagonally, separating the basins of attraction of the two options into almost identical halves with a slight preference for option A, due to the slightly higher input current. In the top right corner, at the intersection of the separatrix and the two nullclines, a high-activity saddle was located that pulled the state diagonally, close to the separatrix, during the first phase of evidence integration (upper row). As feedback from PFC continued to increase the phase portrait reconfigured (middle row): the separatrix moved to the top left, enlarging the basin of attraction of the correct option, and increasing flow towards the correct option. Time to integrate evidence is limited in this scenario: the two high activity attractors enforce a decision after a certain amount of time, as the state of the system is inevitably pulled towards one attractor. The more time that had elapsed, and the farther away from the separatrix the state was already pulled, the less was it possible for the circuit to integrate further evidence before a decision was finally taken. Consequently, the system jumps towards a conclusion that was essentially formed during an early and narrow time window of the decision-making process, which increases the possibility for random rather than sensory inputs to trigger the decision. Therefore, unrelated background activity or noisy evidence during the initial phase of the decision-making had a decisive impact on the evolution of the system in later stages and determined the decision already early in the evidence integration process. The situation was considerably different for the decreased input amplitude because in this scenario the high-activity saddle was located closer towards the origin, and flow towards the attractors was

reduced (2nd column). The decreased flow kept both populations in a low firing state close to the separatrix over a longer amount of time, which yielded more opportunity to take evidence into account that arrived later. For the scenario with the lowest input amplitude a bifurcation had split the saddle into two saddles and a new stable attractor had appeared (1st column). This created a third basin of attraction that kept the state of both PPC populations within a low-firing regime, which enabled to hold up the decision and to continue integrating evidence over a longer time. In this scenario, flow was more diagonally oriented towards the new low-activity attractor and only after reconfiguration of the phase portrait due to PFC feedback (middle row) the system's states moved on to converge towards one of the high activity attractors. Importantly, the more the input amplitude was reduced the more did the decision-making depend on recurrent excitation from PFC. While in the higher-amplitude scenarios (columns three to five) the ramping of PFC towards a decision attractor was driven by ramping in PPC this contrasts with the lower-amplitude scenarios (columns one and two) where PFC had ramped earlier and drove the ramping in PPC (compare how in the first and second panels the PFC population is first to increase towards the high firing rate, while in the third, fourth and fifth panels the PPC population increases first, followed by PFC). That is, in the lower-amplitude scenario recurrent connectivity in the frontoparietal loop had reverberated and integrated evidence over a wider time scale, which led to a higher percentage of correct decisions. These dynamics suggest a cognitive mechanism that gives high-level prefrontal regions control to stall on decisions for extended amounts of time, creating the opportunity to integrate sufficient evidence for making more complex decisions. In contrast, in the higher-amplitude scenarios the PPC populations had the ability to ramp activity more autonomously, requiring less feedback from PFC, and providing reduced opportunity for such slowed integration of evidence. Rather, the system was poised to perform quick decisions, as there was high-velocity flow towards the high-activity fixed points. For very high input amplitudes (fifth column) a bifurcation split the saddle into three high activity fixed points: two saddles and a new attractor emerged, which led to rapid ramping of both PPC populations upon stimulation. This fast ramping enabled rapid decision-making: the system quickly jumped to a high activity state which enabled quick reaction to sensory inputs. As visible in the last column of Supplementary Fig. 4b, the emerging high-activity attractors can be interpreted as an undecided-but-anticipating state that keeps both populations active to react to incoming stimuli more quickly, like tennis players performing small left and right movements to react more effectively to an impeding serve instead of staying static (see also Supplementary Movie 1 for phase space animations).

How input correlation modulates DM performance

We analyzed simulation results to better understand why correlations between synaptic inputs led to better DM performance (Fig. 5c, d). Dynamical systems often use noise to mimic the combined perpetual action of fluctuations that originate from variables that are not directly modelled by the deterministic equations of motion. In contrast to other types of noise, like observation noise that merely amounts to a blurring of signals, in stochastic dynamical systems noise acts as a driving force that drastically modifies the deterministic dynamics, leading to complex non-trivial effects that cannot be predicted by considering the deterministic part of the system alone[85,86]. Importantly, we found that noise correlations had a considerable impact on system dynamics that are not visible in traditional phase portraits. An important difference to deterministic systems is that the flow at each point in phase space is not necessarily confined towards a single direction. Rather, noise can potentially move the state also in other directions, depending on the properties of the noise process. While deterministic systems yield a unique gradient vector at each point in phase space, in stochastic systems the gradient vector's orientation and magnitude depend also on the noise term and can therefore vary when the gradient is computed repeatedly. Therefore, vector fields with simple arrows are not suitable to visualize the expected flow into different directions. We therefore extended traditional phase portraits using ellipsoid glyphs: the magnitude of flow in each direction is mapped onto the radius of the glyph at that orientation (Supplementary Fig. 5 and Supplementary Movie 1). For example, if the glyph is a circle, then the magnitude of flow is equal in every direction (isotropic), which indicates a strong impact of noise. Stretched ellipsoids, on the other hand, indicate that flow is stronger into the elongated directions (anisotropic), which indicates a strong deterministic component and a reduced impact of noise. To construct such stochastic phase portraits (SPPs) we computed the gradient at each visualized point 10,000 times for different noise inputs and then used Kernel Density Estimation to

obtain a smoother estimate. The probability density at each location is then turned into a glyph by setting the radius for each orientation proportional to its corresponding probability density. We constructed SPPs for five different settings of noise correlation, keeping everything else fixed, and found considerable differences in their geometries that help explain why increased noise correlation led to higher accuracy but slower decisions. First, we looked at a global feature of this phase portrait: the average flow speed, that is, the average magnitude of the gradient vector for different noise correlations (Supplementary Fig. 7). We found that flow speed follows a U-shape with a minimum at intermediate correlation values ($r \sim 0.65$) and maxima for $r = 0$ and $r = 1$. This aligns with the shape of integration time for different noise correlations, which follows a reversed pattern that peaks for intermediate values and is lowest for zero and full correlation (Fig. 5d): flow speeds are highest for low and high noise correlations and therefore integration times are lowest at those points. When examining the geometries of the five SPPs, an important change of glyph shapes was visible: with increasing noise correlations, flow orientation distributions shifted from uniform to peaked (Supplementary Fig. 6). To quantify the change of glyph shapes, we computed the entropies of the orientation distributions. Glyphs in the low-correlation SPPs had a higher entropy compared to the high-correlation SPPs: the high-entropy circularly shaped glyphs indicate that there was a higher uncertainty regarding the gradient's direction, and a higher impact of random movements on evidence integration, compared to the oval glyphs. Also, for low correlations flow is predominantly oriented horizontally and vertically towards the high activity attractors. In contrast, for high correlations flow is more diagonally oriented, parallel to the separatrix, and tangential to the decision attractors. Crucially, high noise correlations yielded a more diagonal movement that kept the state closer to the separatrix, which kept both options open for continuous integration of evidence. Conversely, low noise correlations come with the tendency to move the state towards of one of the two attractors, which makes it increasingly unlikely to cross back over the separatrix again and to reverse an upcoming decision. This can be explained by comparing the effects of uncorrelated versus correlated noise. Increased correlation implies that moment-to-moment inputs are more similar in magnitude and sign, which means that the system moves in a more coherent (that is, diagonal) way in phase space. That is, with higher input correlation the activities of both PPC populations had a stronger tendency to jointly increase or to jointly decrease. The resulting diagonal movement allowed the system to stay closer to the separatrix, which allowed to continue integrating evidence. Conversely, uncorrelated inputs imply a stronger tendency to move along horizontal and vertical directions, leading the system away from the diagonal and deeper into one basin of attraction, making it more unlikely to integrate further evidence that could allow the system to again reach the other basin of attraction. This mechanism is especially relevant for hard decisions that are based on ambiguous evidence, where decision performance is close to the chance level and factors like noise or brain state play an increasingly important role for the decision outcome. In summary, the degree of correlation between inputs allowed to trade decision-making accuracy with decision-making speed. Furthermore, noise correlations had tremendous impact on system dynamics that were not apparent with conventional phase portraits but became visible in SPPs, which may therefore be relevant for a wide class of models that make use of noise.

How FIC modulates DM

To study the effect of E/I-ratios on DM in isolation we coupled the DM circuit with the two-node model that we used before to create Fig. 4c-j (Supplementary Fig. 9). We found that only when FIC was active (black dots) that the fraction of correct decisions and the time to decision both increased when E/I-ratios were increased (Supplementary Fig. 9a, b). Conversely, when FIC was inactive (blue dots), the fraction of correct decisions and the time to decision both decreased when E/I-ratios were increased. Consequently, only when FIC was active the empirical direct relationships between DM and FC (Fig. 4e, f) were reproduced (Supplementary Fig. 9c, d). Summarizing, the empirically observed relationship between DM performance and FC only emerged when FIC was active.

Supplementary Figures

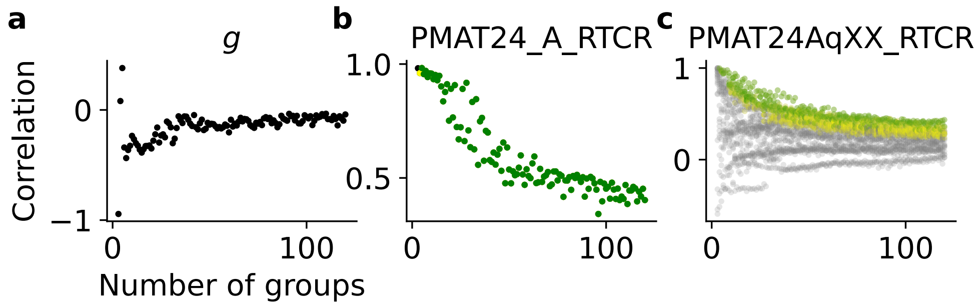

Supplementary Fig. 1 | Correlations between group-average cognitive scores and mean FC for different groupings (three to 120) for all N=650 models. a Correlations between *g*-factor and mean FC. **b** Correlations between PMAT24_A_RTCR and mean FC. **c** Correlations between PMAT24AqXX_RTCR and mean FC. Each dot in panel c represents the correlation between mean FC and PMAT24AqXX_RTCR for each of the 24 PMAT questions for the respective grouping. Obtained p-values of two-sided Pearson's correlation test: yellow dots: $p < 0.05$, green dots: $p < 0.01$; including only p-values that remained significant after controlling for multiple comparisons using the Benjamini-Hochberg procedure with a False Discovery Rate of 0.1.

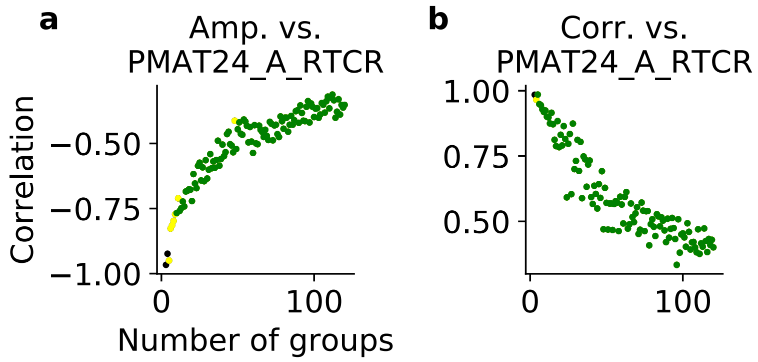

Supplementary Fig. 2 | Correlations between group-average PMAT24_A_RTCR and whole-brain input amplitude, respectively input correlation for different groupings (three to 120) for all N=650 models. a PMAT24_A_RTCR versus input amplitude. **b** PMAT24_A_RTCR versus input correlation. Obtained p-values of two-sided Pearson's correlation test: yellow dots: $p < 0.05$, green dots: $p < 0.01$; including only p-values that remained significant after controlling for multiple comparisons using the Benjamini-Hochberg procedure with a False Discovery Rate of 0.1.

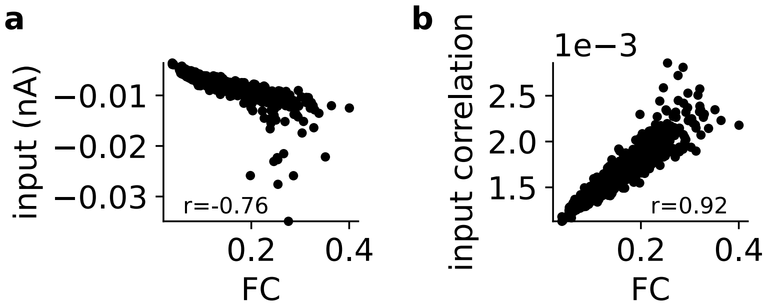

Supplementary Fig. 3 | Empirical FC correlates with simulated synaptic currents on a single-subject level for all N=650 models. **a** Mean whole-brain synaptic current amplitudes decreased with mean FC (r=-0.76, $p = 2.0 \times 10^{-137}$). **b** Mean whole-brain correlation of synaptic currents increased with mean FC (r=0.92, $p = 2.0 \times 10^{-305}$). Obtained p-values remained significant after controlling for multiple comparisons using the Benjamini-Hochberg procedure with a False Discovery Rate of 0.1.

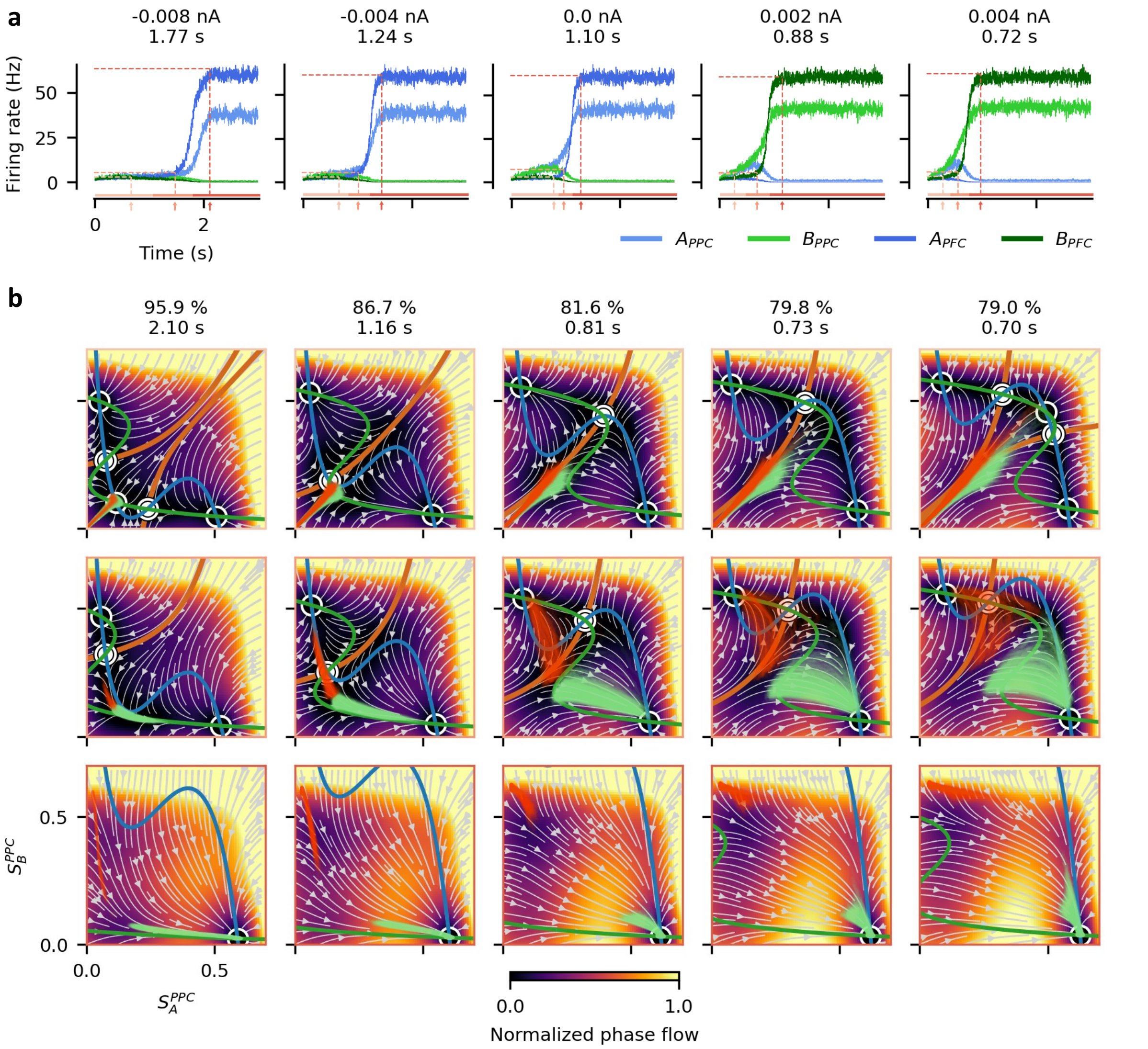

Supplementary Fig. 4 | Input amplitude switches between fast-but-faulty and precise-but-slow DM. a exemplary firing rate time series of all four PPC and PFC populations for five simulations that only differed in input amplitude. In the example, correct decisions were made for the three smaller input offsets and wrong decisions were made for the two larger offsets (titles indicate offset and time until decision). Arrows indicate time points of interest related to three different levels of feedback from PFC. **b** Phase space plots for the three TOIs (from top to bottom) and the five input offset scenarios (from left to right, values as in a). Titles indicate percent correct decisions and average decision time for each offset over 1000 simulations with different noise time series. X-axes: synaptic activity of PPC population A (correct decision); y-axes: synaptic activity of PPC population B (wrong decision). Phase space background colours denote the speed and arrows the direction of flow. Blue and green lines depict nullclines of A and B, respectively. Circles with black faces indicate attractors, circles with white faces indicate saddles. Brown lines show separatrices. The semi-transparent green and red lines depict trajectories around the time points of interest that ended with correct and incorrect decisions in the 1000 test simulations.

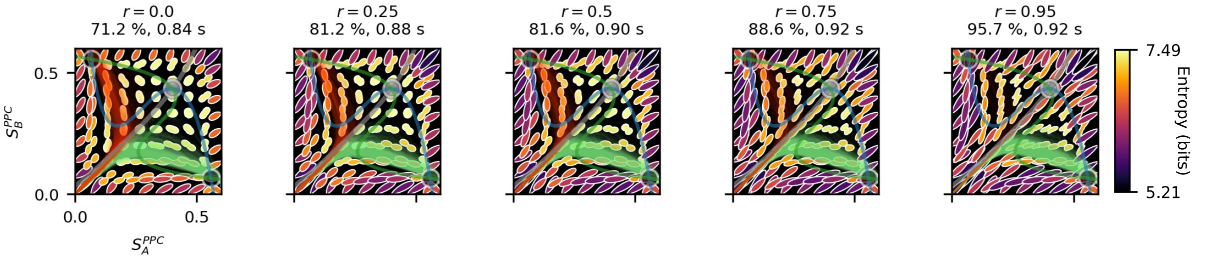

Supplementary Fig. 5 | Stochastic phase portraits for five different correlations of input noise to the populations A and B in the PPC module $I_{noise,A}^{PPC}$ and $I_{noise,B}^{PPC}$ (Eq. 10). Due to noise the flow at each location is not confined to the single direction and magnitude given by the deterministic component of the dynamic system. Stochastic flow is visualized using ellipsoid glyphs where the radius at each orientation is proportional to the magnitude of flow in this direction. Circular glyphs indicate isotropic flow while ovals indicate anisotropic flow. Glyphs were generated by computing 10000 gradients of the model's differential equations at each location for five noise correlation settings (indicated in titles, along with average decision-making accuracies and integration times). Colors indicate the entropy of the underlying

distributions with a higher entropy indicating more isotropic flow. With increasing noise correlations, the shape of the flow distributions gets less uniform and more peaked, which leads to increasingly diagonal flow (note how entropy, especially along the diagonal, increases from left to right). Diagonal flow prolongs the winner-take-all race allowing to integrate more evidence. Conversely, the prominent horizontal and vertical flows for lower correlations pull the system towards one of the two attractors, which makes it increasingly unlikely that the state can move back over the separatrix again.

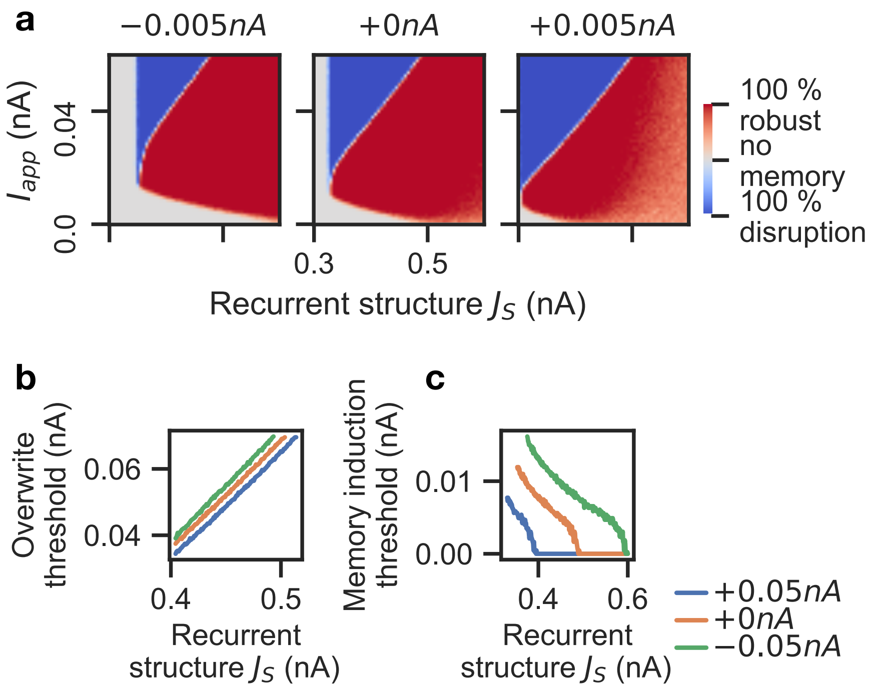

Supplementary Fig. 6 | Effect of input amplitude on WM in the isolated DM circuit. a Bifurcation diagram showing robustness of WM as a function of strength of recurrent structure J_S (recurrent excitation minus local inhibition) and stimulus strength I_{app} for three different mean input amplitudes. Red: WM was not distracted by novel stimuli; blue: novel stimuli were strong enough to disrupt WM; gray: stimulus was too weak to induce WM. Bifurcations shifted depending on mean input amplitude. **b** Decreased mean input amplitude increased the threshold for WM to be overwritten, while increased input decreased this threshold. **c** Decreased mean input amplitude increased the threshold for induction of memory states, while increased inputs made it easier for stimuli to trigger new WM states.

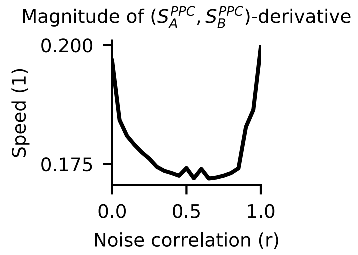

Supplementary Fig. 7 | Average flow speed for different noise correlations in Supplementary Fig. 5.

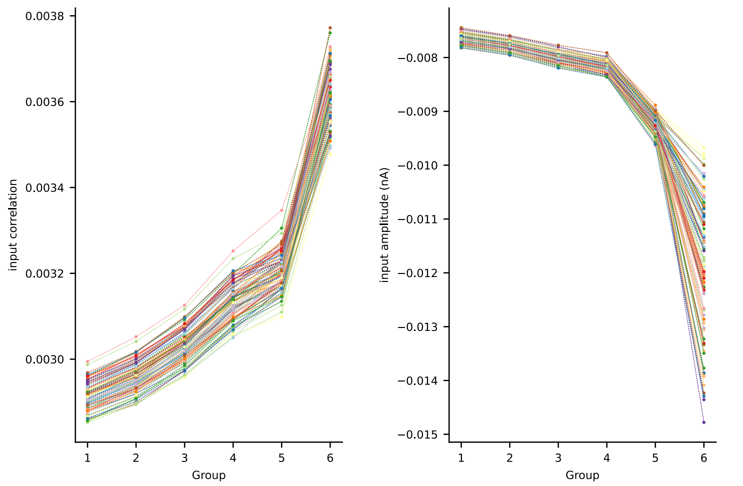

Supplementary Fig. 8 | Model Validation. Subjects were divided into six groups according to their PMAT24_A_RTCR to test whether two key model predictions related to DM performance (amplitude of synaptic inputs decreases and correlation increases for increasing PMAT24_A_RTCR) are robustly reproduced over different fitting runs. For each group, group-average SC and FC were computed and the corresponding model was fitted 100 times using each time a different random noise generator seed and different initial conditions for the six groups. In all 100 tests input correlation monotonically increased (left panel) and input amplitudes monotonically decreased (right panel) from low to high PMAT24_A_RTCR, which confirmed that these key model predictions are robustly produced independent of the fitting run.

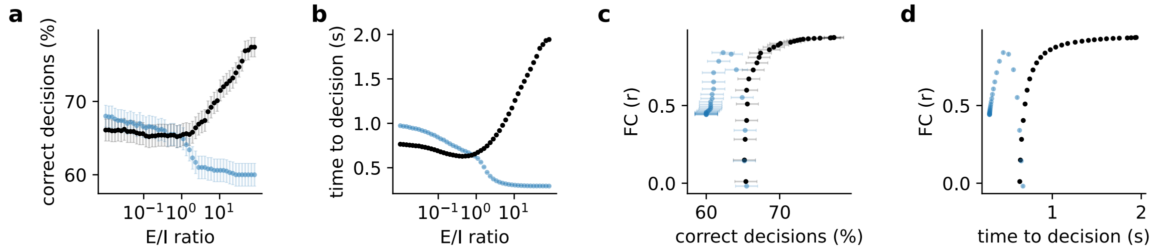

Supplementary Fig. 9 | DM performance depends on the E/I-ratios of synaptic inputs, enabling to trade decision-making accuracy with speed. With active FIC (active FIC shown in black, inactive FIC in blue) the relationship between FC and DM performance reflected the empirically observed relationship (higher FC associated with longer time to decision, Fig. 1f-h). a E/I-ratio versus percent correct decisions. **b** E/I-ratio versus time to decision. **c** Percent correct decisions versus FC. **d** Time to decision versus FC. Data are presented as mean values +/- SD derived over 1000 simulations with different random number generator seeds.

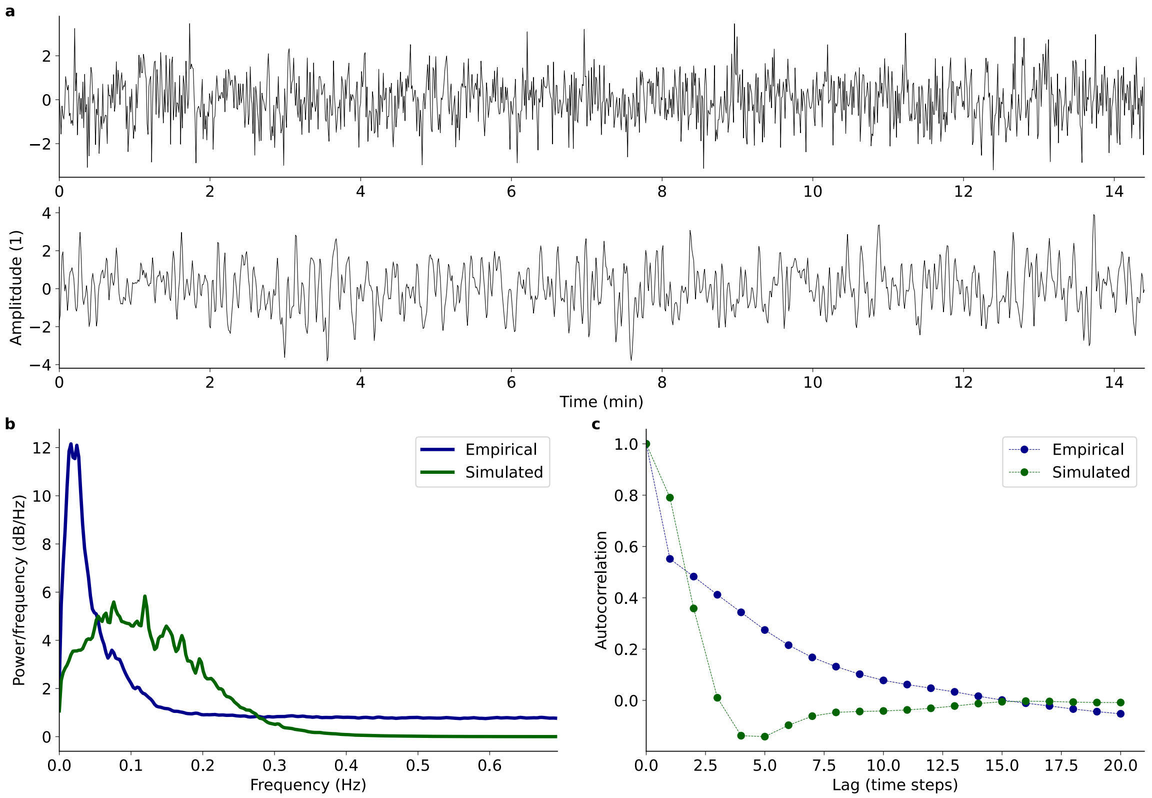

Supplementary Fig. 10 | Empirical versus simulated dynamics. a Exemplary empirical (upper panel) and simulated time series (lower panel). **b** Average power spectral density over six randomly selected subjects (Empirical) in comparison to the average PSD of the six groups from the reproduction test (Simulated, please see section Model validation). **c** Average autocorrelation of the time series data from panel b.
