# Supplementary material for: Learning how network structure shapes decision-making for bio-inspired computing
Source: Nat Commun. 2023 May 23;14:2963. doi: 10.1038/s41467-023-38626-y (PMC10206104; doi:10.1038/s41467-023-38626-y)
Supplement: Supplementary file 3 — Description of Additional Supplementary Files [file 41467_2023_38626_MOESM3_ESM.pdf]

Description of Additional Supplementary Files Document

Supplementary Movie 1. - Overview over the modelling approach and the main results.
