# Supplementary material for: Learning how network structure shapes decision-making for bio-inspired computing
Source: Nat Commun. 2023 May 23;14:2963. doi: 10.1038/s41467-023-38626-y (PMC10206104; doi:10.1038/s41467-023-38626-y)
Supplement: Supplementary file 5 — Reporting Summary [file 41467_2023_38626_MOESM5_ESM.pdf]

nature portfolio

Corresponding author(s): Michael Schirner and Petra Ritter

Last updated by author(s): Apr 27, 2023

Reporting Summary

Nature Portfolio wishes to improve the reproducibility of the work that we publish. This form provides structure for consistency and transparency in reporting. For further information on Nature Portfolio policies, see our Editorial Policies and the Editorial Policy Checklist.

Statistics

For all statistical analyses, confirm that the following items are present in the figure legend, table legend, main text, or Methods section.

n/a	Confirmed	
☐	☒	The exact sample size (*n*) for each experimental group/condition, given as a discrete number and unit of measurement
☒	☐	A statement on whether measurements were taken from distinct samples or whether the same sample was measured repeatedly
☐	☒	The statistical test(s) used AND whether they are one- or two-sided<br>*Only common tests should be described solely by name; describe more complex techniques in the Methods section.*
☐	☒	A description of all covariates tested
☐	☒	A description of any assumptions or corrections, such as tests of normality and adjustment for multiple comparisons
☐	☒	A full description of the statistical parameters including central tendency (e.g. means) or other basic estimates (e.g. regression coefficient) AND variation (e.g. standard deviation) or associated estimates of uncertainty (e.g. confidence intervals)
☐	☒	For null hypothesis testing, the test statistic (e.g. *F*, *t*, *r*) with confidence intervals, effect sizes, degrees of freedom and *P* value noted<br>*Give P values as exact values whenever suitable.*
☒	☐	For Bayesian analysis, information on the choice of priors and Markov chain Monte Carlo settings
☒	☐	For hierarchical and complex designs, identification of the appropriate level for tests and full reporting of outcomes
☐	☒	Estimates of effect sizes (e.g. Cohen's *d*, Pearson's *r*), indicating how they were calculated

Our web collection on statistics for biologists contains articles on many of the points above.

Software and code

Policy information about availability of computer code

Data collection	No software was used for data collection in the current study.
Data analysis	Commercial: MATLAB version R2020a<br>Open Source: Python version 3.9.7 and multiple Python packages (scipy 1.7.1; numpy 1.20.3; matplotlib 3.4.3; scikit-learn 1.1.3; statsmodels 0.12.2; scikit-posthocs 0.7.0); GCC 9.4; FreeSurfer v7.1.0; MRtrix3 3.0; FSL 6.0<br>Custom: implemented using Python, C and MATLAB languages and published at https://github.com/BrainModes/fast-slow/

For manuscripts utilizing custom algorithms or software that are central to the research but not yet described in published literature, software must be made available to editors and reviewers. We strongly encourage code deposition in a community repository (e.g. GitHub). See the Nature Portfolio guidelines for submitting code & software for further information.

Data

Policy information about availability of data

All manuscripts must include a data availability statement. This statement should provide the following information, where applicable:

- Accession codes, unique identifiers, or web links for publicly available datasets
- A description of any restrictions on data availability
- For clinical datasets or third party data, please ensure that the statement adheres to our policy

All data used in this study was derived from the Human Connectome Project Young Adult study available in the repository https://db.humanconnectome.org/data/

projects/HCP_1200. The derived data generated in this study are available under restricted access due to data privacy laws, access can be obtained within a timeframe of one month from the corresponding authors M.S. and P.R. as processing and sharing is subject to the European Union General Data Protection Regulation (GDPR), requiring a written data processing agreement, involving the relevant local data protection authorities, for compliance with the standard contractual clauses by the European Commission for the processing of personal data under GDPR (https://commission.europa.eu/publications/standard-contractual-clauses-controllers-and-processors-eueea_en). The data processing agreement and dataset metadata are available in EBRAINS (https://search.kg.ebrains.eu/instances/88507924-8509-419f-8900-109accf1414b).

Human research participants

Policy information about studies involving human research participants and Sex and Gender in Research.

Reporting on sex and gender	The HCP study population consisted of 497 female and 434 male participants (Wu et al. 2022, NeuroImage) obtained by giving each participant a number of demographic questions including gender (https://www.humanconnectome.org/storage/app/media/documentation/s1200/HCP_S1200_Release_Reference_Manual.pdf). The subset of 650 subjects with complete data and no quality control issues consisted of 360 female and 290 male participants. No sex- or gender-based analyses where performed as our goal in this first explorative study was to elucidate mechanisms that are independent of sex or gender.
Population characteristics	The HCP Young Adult population consists of healthy young adults with mean age of 28.81±3.70 (mean+/-SD)(Wu et al. 2022, NeuroImage).
Recruitment	According to HCP documentation during the HCP recruitment process "White non-Hispanic, Hispanic, Asian and African-American families will be invited to participate, to reflect the ethnic diversity of America" (https://www.humanconnectome.org/study/hcp-young-adult/project-protocol/recruitment)
Ethics oversight	Washington University in St. Louis Institutional Review Board (IRB); Medical Ethical Committee of the Charité Medical Center in Berlin (EA4/184/20).

Note that full information on the approval of the study protocol must also be provided in the manuscript.

Field-specific reporting

Please select the one below that is the best fit for your research. If you are not sure, read the appropriate sections before making your selection.

☒ Life sciences ☐ Behavioural & social sciences ☐ Ecological, evolutionary & environmental sciences

For a reference copy of the document with all sections, see nature.com/documents/nr-reporting-summary-flat.pdf

Life sciences study design

All studies must disclose on these points even when the disclosure is negative.

Sample size	Every HCP Young Adult subject with complete MRI and behavioral data that surpassed the quality control criteria was used for the study (N=650).
Data exclusions	Analysis was restricted to N=650 subjects with complete MRI data including all four sessions of resting-state fMRI, structural MRI (T1w and T2w), diffusion-weighted MRI (dwMRI) as well as the behavioral measures 'PMAT24_A', 'CardSort', 'ProcSpeed' and 'Flanker' were available. In addition, all subjects that were identified by HCP with Quality Control issues were excluded: https://wiki.humanconnectome.org/pages/viewpage.action?pageId=88901591. One additional subject was excluded because there were absent connections in the structural connectome that was more than four standard deviations from the mean over all subjects. The first two reasons were pre-established, while the one subject with spurious brain connectivity was only detected during the processing.
Replication	All attempts at replication were successful and are described in the Results section "Model validation". The fitting procedure was performed 1000 times with random initial conditions and noise generator seeds using the average SC/FC. In addition, group-average SC/FC for six groups models were fitted each 100 times with random initial conditions and noise generator seeds. Regression model fitting to associate inferred with behavioral parameters were repeated 1000 times using different random train and test groups.
Randomization	No experimental conditions requiring randomization were applied in the study.
Blinding	No procedures requiring blinding were applied in the study

Reporting for specific materials, systems and methods

We require information from authors about some types of materials, experimental systems and methods used in many studies. Here, indicate whether each material, system or method listed is relevant to your study. If you are not sure if a list item applies to your research, read the appropriate section before selecting a response.

Materials & experimental systems

n/a	Involved in the study
☒	☐ Antibodies
☒	☐ Eukaryotic cell lines
☒	☐ Palaeontology and archaeology
☒	☐ Animals and other organisms
☒	☐ Clinical data
☒	☐ Dual use research of concern

Methods

n/a	Involved in the study
☒	☐ ChIP-seq
☒	☐ Flow cytometry
☐	☒ MRI-based neuroimaging

Magnetic resonance imaging

Experimental design

Design type: resting-state

Design specifications: 4x 15 minute recordings of resting-state activity.

Behavioral performance measures: No behavioral measures were acquired during the resting-state recordings.

Acquisition

Imaging type(s): functional, structural, diffusion

Field strength: 3

Sequence & imaging parameters: fMRI: Gradient-echo EPI, TR: 720 ms, TE: 33.1 ms, flip angle: 52 deg, FOV: 208x180 mm (RO x PE), Matrix: 104x90 (RO x PE), Slice thickness: 2.0 mm; 72 slices; 2.0 mm isotropic voxels, Multiband factor: 8, Echo spacing: 0.58 ms, BW: 2290 Hz/Px

dwMRI: Spin-echo EPI, TR: 5520 ms, TE: 89.5 ms, flip angle: 78 deg, refocusing flip angle: 160 deg, FOV: 210x180 (RO x PE), matrix: 168x144 (RO x PE), slice thickness: 1.25 mm, 111 slices, 1.25 mm isotropic voxels, Multiband factor: 3, Echo spacing: 0.78 ms, BW: 1488 Hz/Px, b-values: 1000, 2000, and 3000 s/mm2

structural: T1w_MPR1, 3D MPRAGE, TR: 2400, TE: 2.14, TI: 1000, Flip angle: 8 deg, FOV (mm): 224x224, voxel size: 0.7 mm isotropic, BW: 210 Hz/Px

Area of acquisition: whole brain

Diffusion MRI: ☒ Used ☐ Not used

Parameters: 3 shells (b=1000,2000,3000 s/mm2) with approx. 90 gradient directions per shell, no cardiac gating

Preprocessing

Preprocessing software: The fully preprocessed data as provided by Human Connectome Project was used, described in Glasser, M. F., Sotiropoulos, S. N., Wilson, J. A., Coalson, T. S., Fischl, B., Andersson, J. L., ... & Wu-Minn HCP Consortium. (2013). The minimal preprocessing pipelines for the Human Connectome Project. Neuroimage, 80, 105-124.

Normalization: The data was brought into HCP's standard grayordinate space (the "HCP multimodal parcellation") using a combination of segmentation and surface-registration algorithms, described in:
Glasser, M. F., Coalson, T. S., Robinson, E. C., Hacker, C. D., Harwell, J., Yacoub, E., ... & Van Essen, D. C. (2016). A multi-modal parcellation of human cerebral cortex. Nature, 536(7615), 171-178.

Normalization template: No template was used; the HCP grayordinate standard space is assigned through a combination of segmentation, surface registration and classification algorithms described in:
Glasser, M. F., Coalson, T. S., Robinson, E. C., Hacker, C. D., Harwell, J., Yacoub, E., ... & Van Essen, D. C. (2016). A multi-modal parcellation of human cerebral cortex. Nature, 536(7615), 171-178.

Noise and artifact removal: The fully preprocessed data as provided by Human Connectome Project was used as described in Glasser, M. F., Sotiropoulos, S. N., Wilson, J. A., Coalson, T. S., Fischl, B., Andersson, J. L., ... & Wu-Minn HCP Consortium. (2013). The minimal preprocessing pipelines for the Human Connectome Project. Neuroimage, 80, 105-124.
HCP's approach for artifact removal includes: readout, EPI, B0 inhomogeneity, eddy current and gradient distortion correction.

Volume censoring: No volume censoring was performed.

Statistical modeling & inference

Model type and settings	Mathematical modelling with parameter optimization via custom algorithm to produce main results. Correlation analysis to estimate dependencies between variables. Associated analyses used a multiple regression model to compute the coefficient of multiple correlation and linear regression models for model validation.
Effect(s) tested	Fit to network topology (FC) using correlation and root-mean-squared error; tests for correlation and normality.

Specify type of analysis: ☐ Whole brain ☐ ROI-based ☒ Both

Anatomical location(s)	Anatomical locations were obtained using HCP's multimodal parcellation: Glasser, M. F., Coalson, T. S., Robinson, E. C., Hacker, C. D., Harwell, J., Yacoub, E., ... & Van Essen, D. C. (2016). A multi-modal parcellation of human cerebral cortex. Nature, 536(7615), 171-178.
Statistic type for inference (See Eklund et al. 2016)	Connectome-level correlation analysis.
Correction	FDR correction for multiple comparisons.

Models & analysis

n/a	Involved in the study
☐	☒ Functional and/or effective connectivity
☐	☒ Graph analysis
☐	☒ Multivariate modeling or predictive analysis

Functional and/or effective connectivity	Pearson correlation
Graph analysis	average node degree in weighted graph (average correlation in FC matrix) on subject- and group-level
Multivariate modeling and predictive analysis	Mathematical brain network model to simulate neural population interaction in a 379-nodes whole-brain network that simulates input currents, firing rates, synaptic activity and fMRI time series for every node.
